# Mechanisms Underlying the Therapeutic Effects of *Brucea javanica* in Cervical Cancer Treatment Based on Network Pharmacology and Molecular Docking

**DOI:** 10.1155/ijog/9956789

**Published:** 2025-05-30

**Authors:** Wen Jin, Bin Li, Lu Zhang, Chenyang Sun, Yiping Liu

**Affiliations:** Department of Gynecology, Shaanxi Provincial People's Hospital, Xi'an, China

**Keywords:** *Brucea javanica*, cervical cancer, drug target analysis, enrichment analysis, molecular docking, PPI

## Abstract

**Aims:** The aim of this study was to systematically analyze the role of *Brucea javanica* in the treatment of cervical cancer (CC) and its underlying mechanisms by means of network pharmacology and molecular docking.

**Background:** *Brucea javanica* is a traditional Chinese herbal medicine used for the treatment of malaria and cancers, but its mechanism of action in CC is unknown.

**Objective:** The objective of the study is screening of active chemical constituents of *Brucea javanica* by Traditional Chinese Medicine Systems Pharmacology (TCMSP) database and investigating their potential targets involved in CC therapy.

**Methods:** The GeneCards database was used for the disease targets of CC, the drug–compound–disease target network was constructed by using the Cytoscape 3.8.0 software. Then, the key targets in the protein–protein interaction (PPI) network were identified, and the “clusterProfiler” was used for the Gene Ontology (GO) and Kyoto Encyclopedia of Genes and Genomes (KEGG) analysis. The qRT-PCR, CCK-8, and flow cytometry were used to assess the expression levels of specific target genes in CC cells, as well as their effects on cell proliferation, apoptosis, and reactive oxygen species (ROS) levels, respectively. Protein–compound complex analysis was performed using molecular dynamics simulation.

**Results:** A total of 15 active compounds and their 86 treatment targets were obtained from the *Brucea javanica* analysis, in which 51 target genes were associated with the CC-related disease targets. Then, a PPI analysis identified 12 key genes (including *EGFR*, *TP53*, *BCL2*, *AKT1*, *JUN*, *TNF*, *CASP3*, *IL6*, *MMP9*, *ERBB2*, *CCND1*, and *PTGS2*) that were related to oxidative stress, PI3K-Akt, IL-17, p53, and JAK-STAT pathways, inflammatory response, and apoptosis pathways. In addition, *AKT1* showed upregulation at the mRNA level in SiHa cells, and the knockdown of *AKT1* significantly reduced the proliferation of CC cells and increased apoptosis and ROS levels. Molecular docking and dynamics simulations revealed a close binding between the active compounds and targets.

**Conclusions:** The present research comprehensively examined the active compounds, potential targets, and pathways of *Brucea javanica* in CC treatment, providing a novel insight for CC treatment.

## 1. Introduction

Cervical cancer (CC) is a common and invasive malignancy [1, 2]; its high morbidity and mortality are growing global burdens to women's health [2], ranking as the third most prevalent cancer death among young women since 2019 [3, 4]. The epidemiologic risk factors of CC include smoking, age, high-risk human papillomavirus (hrHPV) infection [5], childbirth, oral contraception, and chronic immunosuppression [6, 7]. Among these risk factors, the persistent hrHPV infection (particularly Type 16 (50% CC cases) and 18 (10% CC cases) that encoded the major oncoproteins E5, E6, and E7 for neoplastic transformation) appears to be the main driver associated with CC development [8], increasing the cancer risk by 435-fold and 248-fold, respectively [9]. Most patients develop precancerous lesions (involving the transformation of normal cervical epithelium to preneoplastic cervical) and further develop CC [10]. According to the annual cancer report, approximately 13,820 new cases of CC were diagnosed, and 4,360 estimated deaths occurred in 2024 [3]. Notably, the incidence of CC has decreased by half since the mid-1970s due to the widespread application of cytological screening and treatment of precursor lesions utilizing the Papanicolaou test [11], and the World Health Organization (WHO) points out that CC is a preventable disease [12, 13]. In particular, the declines have accelerated in the youngest population, who received the carcinogenic HPV vaccine that was first approved by the Food and Drug Administration (FDA) for use in 2006 [14]. For instance, the incidence of invasive CC in 20–24-year-old women decreased by 65% in 2012–2019 compared with 24% in 2005–2012 [3]. However, the decline of these decades has reversed in 30–44-year-old women; the incidence rate increased by 1.7% per year in 2012–2019 [3], and locally advanced CC accounts for roughly 32% of all patients, with 40%–50% 5-year survival rate despite conventional treatment applications [15] and metastatic CC is incurable [16]. Therefore, new therapeutic approaches are needed.

*Brucea javanica* (L.) Merr. is in the Simaroubaceae family and is distributed widely in tropical and subtropical zones of China (Fujian, Yunnan, and Guangxi provinces) [17]. Its dry and ripe fruits can be used for medicinal use, that is, Fructus Bruceae, which is commonly called Ya-Dan-Zi in China [18]. The first record as Chinese medicine is in the Compendium of Materia Medica Omission (Ben-Cao-Gang-Mu-Shi-Yi) in the Qing Dynasty [19]. In clinical practice in China, Fructus Bruceae is commonly used for dysentery and malaria treatment [20]. The Chinese Pharmacopoeia recorded its drug properties, including diarrhea, malaria, intestinal inflammation, and several cancer treatments [19]. In addition, *Brucea javanica* is also useful for ulcers, hemorrhoids, hyperkeratosis, and abdominal pain [21], as well as for topical treatment of warts and corns disease [22]. Phytochemical investigations revealed that oleic acid, linoleic acid, tetracyclic triterpene quassinoids, olein, pregnane glucosides, sesquiterpenes, and anthraquinones are the primary components in *Brucea javanica* fruits [23, 24]. In particular, *Brucea javanica* oil (BJO) containing many medicinally active compounds (quassinoids and fatty acid) was developed as a single plant-based Chinese patent medicine [25], in which the patented products, that is, BJO soft capsule and BJO emulsion injection, have been widely used in solid tumor chemotherapy conjunction [26]. Its main anticancer actions include immune boosting, anti-inflammation, and modulation of gut microbiota [18, 26]. Isolated active compounds from *Brucea javanica* had other biological properties, such as anti-inflammatory, cytotoxic, and antiviral activities [27, 28], which encouraged the exploration of chemical components of *Brucea javanica* in cancer treatment [29]. Meanwhile, *Brucea javanica* exhibited cancer inhibition potential in CC cell assay [30], but its exact mechanism, including potential targets and biological processes (BPs), has not been fully elucidated.

Traditional Chinese medicine (TCM) involves multiple pathological targets and pathways in disease treatment [31]. Accordingly, the traditional pharmacology approaches exist enormous limitations in mechanistic studies of TCM [32]. The “network pharmacology” provides novel insight for determining the TCM mechanisms of action; TCM network pharmacology involves network database retrieval [33], bioinformatic network construction and virtual computing analysis, high-throughput data analysis, and network topology [34, 35]. This method focuses on multitarget, multicomponent, and multichannel synergy and is well suited for TCM analyses [36, 37]. The merging of network pharmacology and bioinformatics has become a robust tool in exploring the underlying BPs of disease and drug effects at the molecular level [38, 39]. The current study explored the major bioactive compounds of *Brucea javanica* and its potential targets and signaling pathways in CC treatment by performing network pharmacology and molecular docking analysis, contributing to the development of developing *Brucea javanica* drug for CC treatment.

## 2. Material and Methods

### 2.1. Screening of Active Compounds and Target of *Brucea javanica*

The *Brucea javanica* was uploaded into TCMSP (http://tcmspnw.com) [40] for the chemical compound analysis; then, the active compounds in *Brucea javanica* were screened by the oral bioavailability (OB) ≥ 30% and drug-like (DL) ≥ 0.18 criterion, and their protein targets were obtained and converted to the corresponding genes through the Universal Protein database (UniProt, https://www.uniprot.org).

### 2.2. Acquisition of CC-Related Targets

The keyword “cervical cancer” was used to screen CC-related targets by using the GeneCards database [41] (https://www.genecards.org), setting a value greater than the median score for target identification and duplicate removal.

### 2.3. Target Prediction of *Brucea javanica* in CC Treatment and the Compound–Target Network Construction

We conducted the intersection analysis [42] between the compound–target of *Brucea javanica* and the disease target of CC to explore the potential interaction target of *Brucea javanica* in CC treatment by using the “ggplot2” and “ggveen” R packages in Venn plot [43]. The Cytoscape 3.8.0 software was further used for drug–compound–disease target network analysis and visualization [44].

### 2.4. Construction and Analysis of PPI

The potential interaction target of *Brucea javanica* was uploaded into the search tool for the retrieval of interacting genes/proteins database (STRING, https://string-db.org/) [45], setting “*Homo sapiens*” as the species and degree of confidence ≥ 0.4 for the PPI network construction. Then, the Centiscape 2.2 plug-in of Cytoscape software was used to analyze PPI topology parameters [44]; the key targets in the PPI network were obtained based on the degree ≥ 40.0 screening condition [46].

### 2.5. Function Enrichment Analysis

The “clusterProfiler” R package was used to perform the GO and KEGG enrichment analysis of *Brucea javanica* targets in CC treatment [47]; the analysis results of cell composition, molecular function (MF), and BP were screened with *p* < 0.05 as the criterion [48].

### 2.6. Molecular Docking

The key target protein structure was obtained from the RCSB Protein Data Bank (RCSB, https://www.rcsb.org/), and the solvent molecules and ligands were removed in Pymol software. Then, the SDF format file of the 2D structure of the active ingredients corresponding to the key targets was downloaded from the TCMSP and PubChem database and was converted into a 3D mol2 format file through the Chem3D software. After that, the AutoDock Tools 1.5.7 software was used to add hydrogen, calculate charge, assign atom types to target proteins, calculate charge, determine torsion center, select torsion bonds, and assign atom types to active compounds. The AutoDock was used for molecular docking analysis [49], and the Pymol software was used for the visualization of receptor ligands [50].

### 2.7. Cell Culture and Transfection

The human normal cervical epithelial cell line Ect1/E6E7 (#CRL 2614) and the SiHa CC cell line (#HTB 35) were obtained from ATCC (Manassas, Virginia, United States). The short tandem repeat (STR) identification has been carried out on the cells, and the outcome of mycoplasma detection shows negativity. All experiments were conducted under the culture conditions at 37°C with 5% CO_2_, utilizing cells that were maintained in Dulbecco's modified Eagle's medium (DMEM) enriched with 10% fetal bovine serum (FBS; Caisson, North Logan, Utah, United States) and 1% penicillin–streptomycin (Caisson). Hereafter, according to the protocol of Lipofectamine 2000 (Invitrogen, California, United States), SiHa cells were transfected with the small interfering (si) RNA of *AKT1* (si-*AKT1*#1: 5⁣′-GACCATGAACGAGTTTGAGTACC-3⁣′; si-*AKT1*#2: 5⁣′-CAGATGGAAAGACGTTTTTGTGC-3⁣′) and negative control (si-NC).

### 2.8. Real Quantitative Real-Time PCR (qRT-PCR)

Total RNA was extracted from SiHa cells using the Trizol reagent extraction kit from Invitrogen. For the synthesis of cDNA, the PrimeScript RT Master Mix kit provided by Takara Bio was utilized. The levels of transcript expression were quantified using SYBR Green qPCR Master Mix (MedChemExpress, Monmouth Junction, New Jersey, United States) on the qPCR Detection System (HealForce, CG-05, Shanghai, China). The experiment was performed in triplicate. Below are the sequences of the PCR primers: *AKT1*, forward 5⁣′-AGCGACGTGGCTATTGTGAAG-3⁣′ and reverse 5⁣′-GCCATCATTCTTGAGGAGGAAGT-3⁣′; GAPDH, forward 5⁣′-TGACTTCAACAGCGACACCCA-3⁣′ and reverse 5⁣′-CACCCTGTTGCTGTAGCCAAA-3⁣′. Relative mRNA expression (AKT1/GAPDH) was determined using the comparative Ct method (2^−*ΔΔ*Ct^).

### 2.9. Cell Proliferation Assay

SiHa cells during the logarithmic growth phase were plated into a 96-well plate with a density of 1 × 10^4^ cells per well and incubated at 37°C with 5% CO_2_ for periods of 0, 24, 48, or 72 h. Subsequently, 10 *μ*L of CCK-8 reagent was introduced to the culture medium, and the samples were allowed to incubate at 37°C for 2 h. For the construction of the CCK-8 curve, the absorbance was measured at 450 nm, which served as the *y*-axis, with time plotted on the *x*-axis. The results were calculated as the average from three independent experiments.

### 2.10. Flow Cytometry for Cell Apoptosis

SiHa cells which were transfected with si-*AKT1* or a negative control were gathered, washed with PBS, and later resuspended in 195 *μ*L of annexin-V FITC (BD Biosciences, Franklin Lakes, New Jersey, United States) mixed with 5 *μ*L of propidium iodide (PI), following the manufacturer's guidelines. After that, flow cytometry was performed after a 10-min incubation of the cells in the dark at room temperature for analysis. The results were assessed using Lysis software (EPICS-XL, Ramsey, Minnesota, United States).

### 2.11. Reactive Oxygen Species (ROS) Detection

To assess the levels of intracellular ROS after the knockdown of *AKT1* in SiHa cells, we utilized DCFH-DA staining coupled with flow cytometry analysis [51]. In brief, SiHa cells during the logarithmic growth phase were subjected to transfection with either si-*AKT1* or si-NC for 48 h. Next, the cells were processed with a working solution of 10 *μ*M 2⁣′,7⁣′-dichlorofluorescein diacetate (DCFH-DA, Sigma, St Louis, Missouri, United States) for 30 min at 37°C in the absence of light and then thoroughly washed with PBS to eliminate any unbound probe. Subsequently, the fluorescence intensity generated by ROS was measured using flow cytometry (FITC channel), and the mean fluorescence intensity (MFI) was calculated to determine the intracellular ROS levels.

### 2.12. Statistical Analysis

R software Version 4.3.2 and GraphPad Prism Version 8.01 were employed in statistical analyses. The data are presented as mean ± standard deviation (SD). Comparisons among multiple groups were executed using either Student's *t*-test or a one-way ANOVA. Each assay was carried out on at least three separate occasions. *p* < 0.05 denoted a statistical significance.

## 3. Results

### 3.1. Identifying Active Compounds and the Disease Target Construction

**Figure 1:**
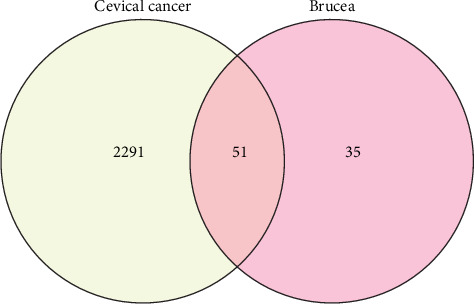
Venn plot of the intersection between the *Brucea javanica* drug targets and the CC disease targets.

**Figure 2:**
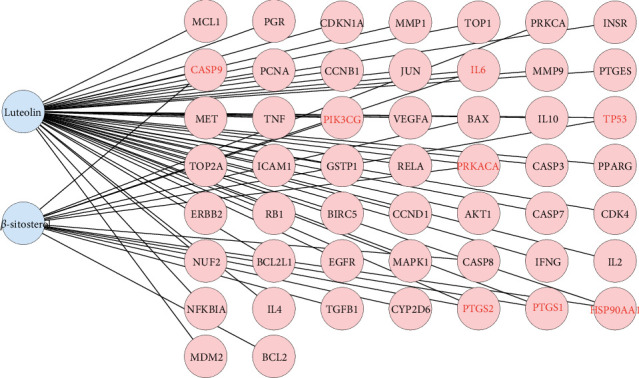
The drug–compound–disease target network analysis.

After TCMSP analysis, we screened 15 active compounds from *Brucea javanica* and their 86 drug targets after eliminating redundancy (Table 1). Subsequently, 2342 disease targets of CC treatment were obtained from 5591 CC-correlated disease retrieval data. The intersection between the *Brucea javanica* drug targets and the CC disease targets determined 51 potential active compound–disease interaction targets (Figure 1), in which luteolin and beta-sitosterol are closely associated with these gene targets in the drug–compound–disease network by using Cytoscape 3.8.0, such as CASP9, PTGS2, PRKACA, PIK3CG, IL6, PTGS1, TP53, and HSP90AA1 that exhibited higher degrees of crosslinking and were regarded as the key nodes (Figure 2). The network provides deep insight into the understanding of the complex effects of *Brucea javanica* active compounds on CC treatment.

### 3.2. Construction and Analysis of PPI

**Figure 3:**
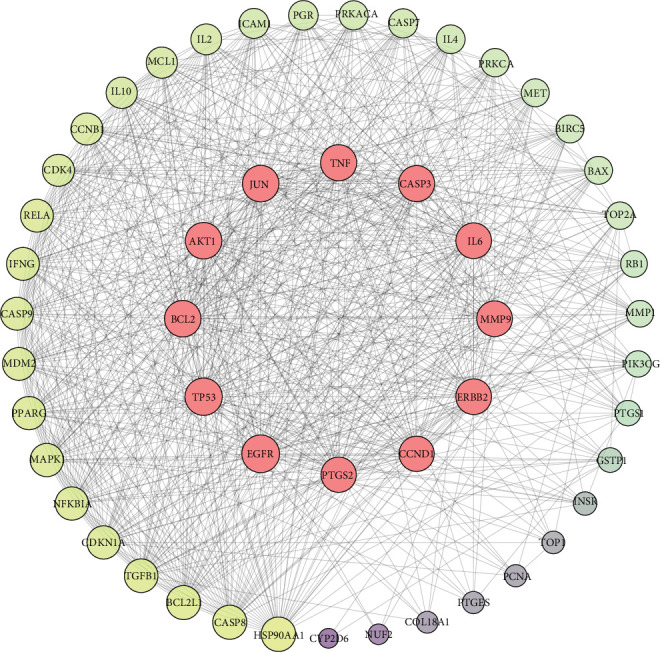
Protein–protein interaction (PPI) network topological analysis.

To investigate the therapeutic effects of *Brucea javanica* against CC, a PPI network was generated by importing these 51 potential active compound–disease interaction targets into the STRING database. In the network, medium confidence > 0.4 was used as the screening criterion for topology analysis, and the top 10 genes were selected as the center genes of the core PPI network. Then, 12 core targets were identified (*EGFR*, *TP53*, *BCL2*, *AKT1*, *JUN*, *TNF*, *CASP3*, *IL6*, *MMP9*, *ERBB2*, *CCND1*, and *PTGS2*), and the network between the core and noncore targets was constructed (Figure 3), in which the target degree is proportional to node sizes. The functions of these core targets play a crucial role in elucidating the biological function of *Brucea javanica.*

### 3.3. GO Enrichment Analysis of Key Target Genes

**Figure 4:**
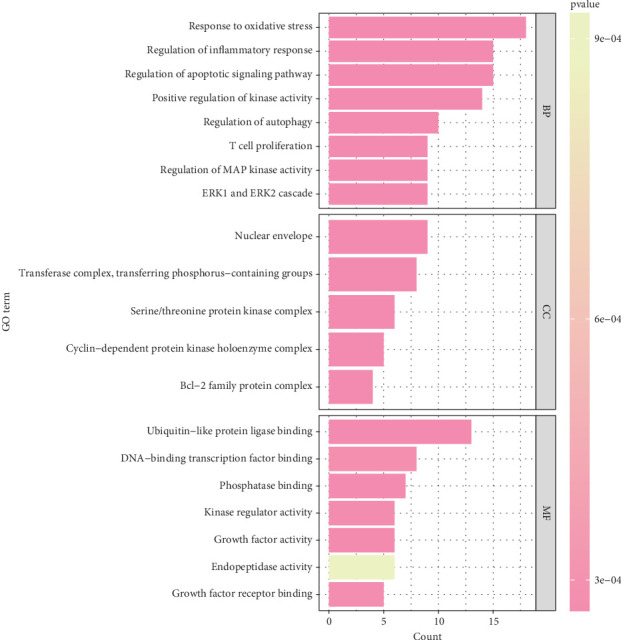
Enrichment analysis of Gene Ontology (GO).

Function enrichment analysis was performed to further explore these 12 key targets. A total of 124 items were identified in three categories, including 32 CC, 71 MF, and 21 BP. The top 10 enriched items related to CC in three categories were shown in bubble charts (Figure 4); the results revealed that these targets are associated with the oxidative stress response, inflammatory regulatory response, apoptosis regulation signaling, positive kinase activity and autophagy, and T cell proliferation pathway in the BP category; associated with the nuclear membrane, transferase complex, transfer phosphorylation, and serine/threonine protein kinase complex pathways in the cell composition category (Figure 4); and associated with the ubiquitination-like protein ligase binding, DNA binding transcription factor binding, and phosphorylation binding pathways in the MF category (Figure 4). These results suggest that *Brucea javanica* may play a multitargeted synergistic therapeutic role in the development of CC by modulating BPs and molecular mechanisms closely related to oxidative stress, apoptosis, and T cell function.

### 3.4. KEGG Enrichment Analysis of Key Target Genes

**Figure 5:**
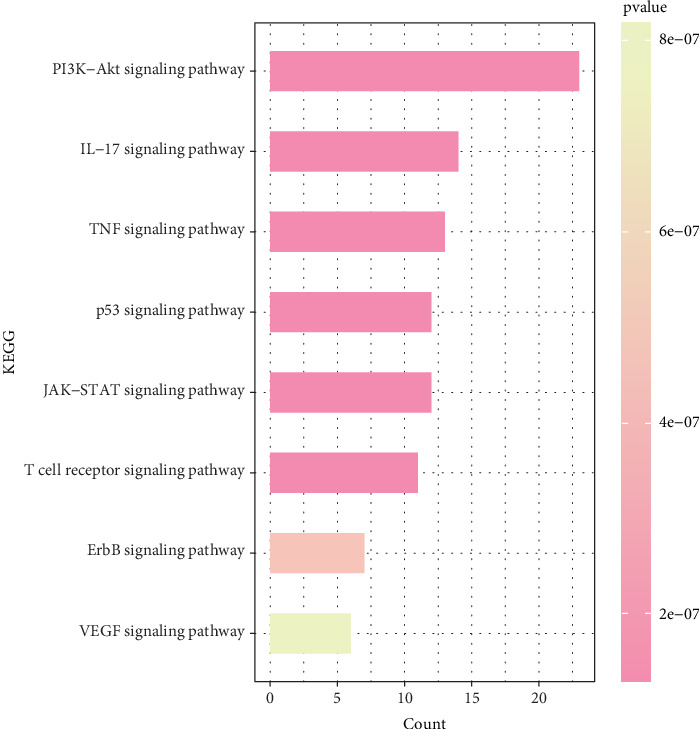
Enrichment analysis of Kyoto Encyclopedia of Genes and Genomes (KEGG).

In order to elucidate the underlying pathway activation of *Brucea javanica* in the treatment of CC, the KEGG enrichment analysis of these targets was performed (*p* < 0.05 as the significance level). The targets were enriched in 171 pathways, in which the filtered CC-related signaling pathways are mainly related to the p53 signaling pathway, TNF signaling pathway, JAK-STAT signaling pathway, IL-17 signaling pathway, PI3K-Akt signaling pathway, etc. (Figure 5). The high enrichment of the PI3K-Akt signaling pathway and IL-17 signaling pathway suggests that these candidate drug molecules may function via these pathways in CC treatment.

### 3.5. AKT1 as a Key Gene to Validate Its Potential Function in CC Cells

**Figure 6:**
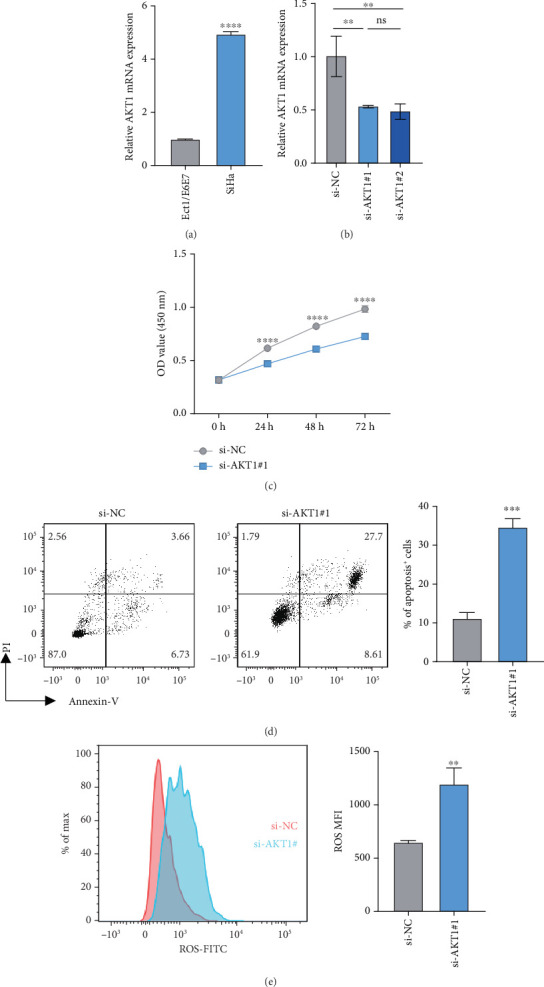
Expression and potential function of the screened key gene *AKT1* in CC cells. (a) Differential analysis of *AKT1* mRNA levels in Ect1/E6E7 cells and SiHa cells. (b) qRT-PCR to verify the efficiency of *AKT1* knockdown in SiHa cells. (c) Effect of *AKT1* knockdown on the proliferative capacity of CC cells. (d) Effect of *AKT1* knockdown on apoptosis levels in CC cells. (e) Effect of *AKT1* knockdown on ROS levels in CC cells. The significance was denoted as either asterisks (“∗”) or “ns.” ∗∗*p* < 0.01, ∗∗∗*p* < 0.001, and ∗∗∗∗*p* < 0.0001, while ns means *p* > 0.05.

To further validate the potential impact of the screened target gene in CC, we selected *AKT1*—a key gene involved in regulating tumor progression and therapy resistance—for functional verification. As shown in Figure 6a, we observed that the mRNA expression level of *AKT1* was significantly upregulated in SiHa cells relative to control cells. Subsequently, we evaluated the potential role of *AKT1* on CC cells by silencing its expression (Figure 6b). CCK-8 assay showed that *AKT1* knockdown markedly inhibited the proliferation of CC cells (Figure 6c). In addition, knockdown of *AKT1* significantly increased the apoptotic ability and ROS level of SiHa cells (Figure 6d,e). These results reveal that *AKT1* gene silencing may induce CC cell apoptosis through enhanced oxidative stress, a mechanism that provides a new theoretical basis for *AKT1* as a therapeutic target.

### 3.6. Molecular Docking Analysis

**Figure 7:**
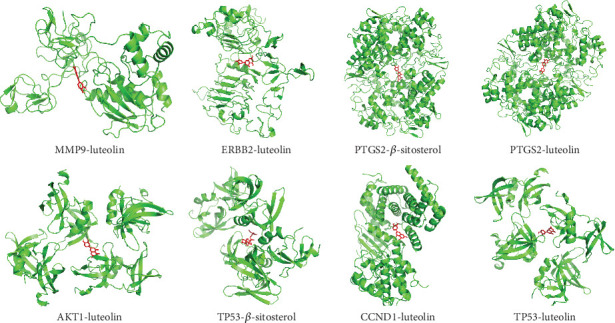
Molecular docking analysis.

Based on the correspondence between the active compounds and the potential target, to validate the network pharmacology findings, the molecular docking analysis of target proteins and active compounds was performed; the spatial coordinates of docking and the details of compounds and targets are shown in Table 2; free binding energies of docking range from −4.37 to −10.53 kcal/mol, suggesting the stable binding. The compound–target interaction results showed that *TP53*–beta-sitosterol, *PTGS2*–luteolin, *PTGS2*–beta-sitosterol, *ERBB2*–luteolin, *CCND1*–luteolin, *TP53*–luteolin, *AKT1*–luteolin, and MMP9–luteolin exhibited the highest free binding energy scores, implying their relatively stable binding ability, and the models are visualized in Figure 7. Notably, molecular docking analysis provided a useful strategy to explore the candidate drug molecules and their disease-associated targets, but the actual effect of these herbal compounds still needs to be verified by experiment.

## 4. Discussion

CC is a frequent malignancy with high mortality in women [52]; screening can reduce the incidence and mortality of this preventable disease [11]. However, unlike some types of cancer data, the survival reports of CC have not shown promising results [53], owing to the symptoms and signs of this cancer (10–15-year latent period of HPV infection [54]) not appearing until an advanced stage [55]. Most CC patients accept standard chemotherapy and radiotherapy; only a few people that received vaccines were cured [56]. Following 2020, the WHO launched a global project to accelerate CC elimination, reducing the irreversible consequences of CC on women and families [57]. Specifically, this study prioritizes vaccination, improved early diagnosis, treatment and detection, expanded research input, and enhanced follow-up of abnormal results. Therefore, the TCM can be a potent potential intervention strategy in the CC elimination project. In our study, we performed a network pharmacology and molecular docking analysis to explore the underlying mechanism of *Brucea javanica* against CC.

Beta-sitosterol (*β*-sitosterol) and luteolin are two main active compounds that respond to target genes of CC. Beta-sitosterol is a subclass of steroids and structurally similar to cholesterol [58]; these phytosterols play an important physiological role and have numerous advantages in eukaryotic organisms, including cholesterol, which is a necessary membrane constituent that affects membrane flexibility and serves as a secondary messenger [59]. *β*-Sitosterol has been shown to have beneficial inflammatory action; animal experiments revealed that the diet combined with 2% *β*-sitosterol can decrease the spread and progression of cancer cells [60]. Low concentrations of *β*-sitosterol can slow cell development and even induce cell death in prostate, murine fibrosarcoma, liver, and breast cancers via various mechanisms [61]. For example, *β*-sitosterol dramatically suppressed G0/G1 phase arrest, Bcl-2 expression, and NF-kB and Akt/GSK-3*β* activity, while increasing Bax expression in pancreatic cancer to prevent cancer progression [62]. The combination of gemcitabine and *β*-sitosterol has been a successful therapy strategy for pancreatic cancer [63]. Luteolin is a natural flavonoid widely present in sweet bell peppers, broccoli, celery, and parsley [64]; the plants rich in luteolin are used in TCM to treat inflammatory disorders, hypertension, and cancer [65]. Modern pharmacological studies revealed that luteolin can hamper the progression of carcinogenesis in metastasis, angiogenesis, invasion, and transformation through multiple mechanisms [66]. In breast cancer, luteolin suppresses the expression of cancer-promoting proteins (p-Akt, p-STAT3, and p-EGFR), reduces progestin-dependent VGF secretion and cell growth viability, and increases Bax expression [67]. In particular, the inhibition of PI3K-Akt, STAT3, and MAPK/Erk1/2 signaling pathways may explain the effect of luteolin as a promising anticancer agent [68]. Luteolin can also protect against lipopolysaccharide-induced acute lung injury in mice and can notably reduce oxidative damage, superoxide dismutase and catalase activities, and lipid peroxidation [69]. Therefore, these two compounds can be hopeful anticancer agents in CC treatment.

Furthermore, the identified 12 key target genes including EGFR, TP53, BCL2, AKT1, JUN, TNF, CASP3, IL6, MMP9, ERBB2, CCND1, and PTGS2 are key target genes in CC treatment. These genes were classified into three categories, including the cancer suppressor genes of TNF, TP53, and IL6 and the oncogenes of JUN, BCL2, AKT1, EGFR2, PTGS2, MMP, CASP3, and CCND1; their exact role in these pathways, including the IL-17 signaling pathway and PI3K-Akt signaling pathway, is worth further study. In addition, it should be noted that this study has some limitations. First, this study mainly relied on public databases such as TCMSP and GeneCards for active compound identification, prediction of targets, and enrichment analysis on pathways, and the results obtained were predictive and theoretical to a certain extent, lacking verification from animal experiments. Subsequent studies will utilize animal models to experimentally validate the screened key targets and pathways in order to enhance the credibility and application value of the results. Secondly, *Brucea javanica* contains a variety of active compounds, which may have synergistic or antagonistic effects in vivo, and the current study only identifies representative components through a network pharmacological approach, which does not yet comprehensively reflect their pharmacological effects in vivo and the interrelationships among components. In this regard, we will further analyze the distribution of the components of the TCM compound in vivo by combining with metabolomics or compositional tracking technology in the future to clarify the main active components and their metabolic behaviors. Finally, our study is currently unable to comprehensively assess the expression trends and prognostic relevance of the screened targets in clinical samples, which also limits the clinical translational potential of the findings. Therefore, subsequent studies will combine the clinical expression profiles and survival analysis from public databases to further validate the association of the predicted targets with the prognosis of CC patients and enhance the clinical applicability of the study.

## 5. Conclusion

To conclude, the molecular mechanisms of *Brucea javanica* in CC treatment were comprehensively explored by performing network pharmacology and molecular docking. Beta-sitosterol and luteolin were identified as the main active compounds of *Brucea javanica* that produced therapeutic effects against CC through activating oxidative stress, inflammatory responses, and apoptosis via several pathways such as JAK-STAT, TNF, p53, and PI3K-Akt signaling pathways. Overall, the mechanism of *Brucea javanica* in CC treatment was analyzed based on a multicomponent and multipathway approach. The current finding may promote the development of a *Brucea javanica* drug strategy in CC treatment.

## Figures and Tables

**Table 1:** The active compounds of *Brucea javanica*.

**Mol ID**	**Molecule name**	**OB (%)**	**DL**
MOL000006	Luteolin	36.16	0.25
MOL000358	Beta-sitosterol	36.91	0.75
MOL008068	Bruceoside A_qt	31.05	0.75
MOL008073	Brusatol	45.69	0.75
MOL008077	Yadanzioside B	46.16	0.31
MOL008089	Yadanzioside H	62.77	0.32
MOL008091	Yadanzioside I	61.13	0.38
MOL008093	Yadanzioside J	38.7	0.3
MOL008097	Yadanzioside L	31.37	0.27
MOL008099	Yadanzioside M	45.04	0.23
MOL008105	Yadanzioside P	58.76	0.29
MOL008108	Yadanziolide C_qt	31.8	0.66
MOL008109	Yadanziolide D	55.76	0.65
MOL008110	Bruceoside B	56.54	0.32
MOL008112	Bruceine C	31.38	0.66

**Table 2:** The molecular docking analysis of target protein and active compounds.

**Gene**	**Entry ID**	**Ingredients**	**Binding energy (kcal/mol)**
PTGS2	3ntg	Beta-sitosterol	−10.53
TP53	8swj	Beta-sitosterol	−6.66
PTGS2	3ntg	Luteolin	−7.6
ERBB2	2a91	Luteolin	−5.79
CCND1	6p8e	Luteolin	−5.6
TP53	8swj	Luteolin	−5.54
AKT1	7wsw	Luteolin	−5.5
MMP9	1l6j	Luteolin	−4.37

## Data Availability

The datasets generated and/or analyzed during the current study are available in the Traditional Chinese Medicine Systems Pharmacology database (http://tcmspnw.com) and GeneCards database (https://www.genecards.org).

## Nomenclature

TCMSP: Traditional Chinese Medicine Systems Pharmacology
CC: cervical cancer
PPI: protein–protein interaction
hrHPV: high-risk human papillomavirus
WHO: World Health Organization
FDA: Food and Drug Administration
BJO: *Brucea javanica* oil
TCM: traditional Chinese medicine
OB: oral bioavailability
GO: Gene Ontology
KEGG: Kyoto Encyclopedia of Genes and Genomes
BP: biological process
MF: molecular function
RCSB: RCSB Protein Data Bank
